# Correlation analysis between micro and macro indicators of high modulus modified asphalt for asphalt pavement

**DOI:** 10.1371/journal.pone.0313820

**Published:** 2025-01-02

**Authors:** Jiarong Wang, Zhengqi Zhang, Zhongnan Tian

**Affiliations:** 1 Shaanxi College of Communications Technology, Xi’an, Shaanxi, China; 2 Key Laboratory for Special Area Highway Engineering of Ministry of Education, Chang’an University, Xi’an, Shaanxi, China; 3 Hebei Provincial Communications Planning, Design and Research Institute Co., Ltd, Shijiazhuang, Hebei, China; Oregon State University, UNITED STATES OF AMERICA

## Abstract

The relationship between the micro technical indexes and the macro road performance of high modulus asphalt (HMA) is helpful for understanding its mechanism and performance, and promoting its application. To explore the relationship, two kinds of high modulus asphalt (HMA), LLDPE/SBS composite modified asphalt and rubber/PPA composite modified asphalt were prepared according to the HMA requirements. Secondly, Molecular models of two kinds of HMA were established through molecular dynamics (MD) simulations, and the high temperature parameters of LLDPE/SBS composite modified asphalt were obtained with the two methods, namely the micro molecular dynamics simulation and high temperature rheological test, respectively. Then, through correlation analysis and regression calculation, the estimation formula was established between the results of molecular dynamics simulation and high temperature rheological test. Finally, in order to evaluate and verify the rationality of the estimation formula, the two methods were carried out on the other HMA (rubber/PPA composite modified asphalt). The results show that the shear modulus obtained by molecular dynamics simulation has a good correlation with the high temperature rheological properties. The estimation formula based on molecular dynamics simulation can be used to estimate the high temperature shear modulus of high modulus asphalt, and the relative error is less than 7%, which means that the formula can be used to effectively predict the high temperature performance of high modulus asphalt.

## 1 Introduction

Due to its good performance characteristics, high modulus asphalt can be used in heavy-load traffic sections and roads with severe rut deformation. Moreover, using high modulus asphalt concrete as pavement base is an important technical requirement for the structural design of long-life pavement. Road experts have done a lot of research and application on high modulus asphalt, and exploration from a micro perspective has also been carried out [1–6]. The current research on the micro layer of high modulus asphalt is mostly limited to qualitative analysis of its modification mechanism through image experiments such as infrared spectrum and scanning electron microscope, and it is difficult to conduct in-depth analysis of the intrinsic mechanism and performance of high modulus asphalt materials. In recent years, molecular dynamics simulation has been gradually used in the field of material performance prediction. Molecular dynamics simulation can be used to analyze the macro performance of polymer modified asphalt under the limited experimental conditions through iterative calculations of the constituent elements, molecular structure, and intermolecular interaction forces of materials. In the study of molecular dynamics of high modulus asphalt, Ding et al. [7] analyzed the effect of SBS on the aggregation behavior of asphalt molecules using radial distribution functions. The results show that the influence level of SBS on the performance of asphalt depends on the length of alkane side chain of asphaltene. Sun et al. [8] calculated the density and glass transition temperature of SBS modified asphalt through molecular dynamics simulation, and The self-healing properties and healing mechanism of SBS modified asphalt were studied by the simulation results of diffusion coefficient and activation energy of asphalt. Feng et al. [9] studied the molecular aggregation state of SBS and asphalt at the molecular level by molecular dynamics simulation. At present, the quantitative analysis of high modulus asphalt performance using molecular dynamics simulation methods are limited, and the relationship between micro technical indexes and the macro road performance of high modulus asphalt seldom investigated. Therefore, it is difficult to predict the performance of high modulus modified asphalt under some extreme test conditions.

If the relationship between the micro technical indexes and the macro road performance of high modulus asphalt can be established, and the performance of high modulus asphalt can be measured from the perspective of molecular dynamics, then the performance of high modulus asphalt can be evaluated under limited experimental conditions. For the purpose, the study is conduced as follows, first, two kinds of high modulus modified asphalt, LLDPE/SBS composite modified asphalt and rubber/PPA composite modified asphalt were prepared according to the technical requirement. The modulus simulation calculation results and high temperature rheological property test results of LLDPE/SBS composite modified asphalt were obtained by micro molecular dynamics simulation method and actual measurement method. Then, the correlation between simulation results and test results were established and analyzed, the appropriate parameters of molecular dynamics simulation were obtained, and the estimation formula between the results of molecular dynamics simulation and high temperature rheological properties was established. Finally, the simulation results and the rheological performance test results of rubber/PPA composite modified asphalt was selected to evaluate and verify the rationality of the estimation formula. Establishing the relationship between microscopic indicators and macroscopic road performance is of great significance for predicting the macroscopic performance of high modulus modified asphalt in the future.

## 2 Materials and preparations

### 2.1 Materials

#### 2.1.1 Base asphalt

SK70 base asphalt used in this study was obtained from Xiamen City, Fujian Province, China. The technical indexes of the base asphalt are listed in Table 1.

**Table 1:** Technical indexes of SK70 base asphalt.

Index	Value
Penetration (25°C, 0.1 mm)	68
Softening point (°C)	48.8
Ductility (15°C, cm)	>150
Retained penetration ratio after RTFOT (%)	67
Retained ductility at 10°C after RTFOT (cm)	6.9

#### 2.1.2 PE modifier

PE modifier used in this study was LLDPE-DFD7042, produced by United Petroleum and Chemical Co., Ltd (Fujian Province, China), which has a good effect on improving the high temperature performance of asphalt. The technical indexes are shown in Table 2.

**Table 2:** Technical indexes of PE.

Index	Value
Density (g·cm^-3^)	0.918
Mass flow rate of solution (190°C, g/10min)	1.8
Elongation at break (%)	766
Tensile strength (MPa)	12.2

#### 2.1.3 SBS modifier

SBS modifier used in this study was Li Changrong 3411 (Star SBS), obtained from Guangzhou City, Guangdong Province, China. The technical indexes are shown in Table 3.

**Table 3:** Technical indexes of SBS.

SBS type	S/B	Volatile matter (%)	Ash content (%)
3411	7/3	0.25	0.1

#### 2.1.4 Rubber modifier

The rubber used in this study comes from the high-grade tire rubber particles with more than 90% natural rubber content produced by Qiangcan rubber and plastic products Co., Ltd (Shanghai City, China). The technical indexes are shown in Table 4.

**Table 4:** Technical indexes of rubber.

Index	Iron content (%)	Fiber content (%)	Relative density	Impurity content (%)
Value	0	0.01	1.19	0.28

#### 2.1.5 PPA modifier

PPA modifier used in this study was No. 80104518, produced by Sinopharm Chemical Reagent Co., Ltd (Shanghai City, China). The technical indexes are shown in Table 5.

**Table 5:** Technical indexes of PPA.

Index	Value
Chloride (%)	≤0.001
Sulfate (%)	≤0.02
Heavy metal (%)	≤0.01
Iron (%)	≤0.01
Phosphorus pentoxide (%)	≥80.0

### 2.2 Preparation of high modulus asphalts

According to the previous research results of the research group, the preparation process of LLDPE/SBS composite modified asphalt and Rubber/PPA composite modified asphalt was determined as follows.

#### 2.2.1 LLDPE/SBS composite modified asphalt

22.5g LLDPE and 22.5g SBS were added to 500 g heated SK70 base asphalt (175 ± 5°C) and swelled for 30 min. Then the mix was sheared at 180°C for 50 min in a high-shear mixer at 4000 rpm/min and developed for 1 hour in an oven at 160°C to ensure the performance of final product.

#### 2.2.2 Rubber/PPA composite modified asphalt

A different method was used to prepare the rubber/PPA modified asphalt specimen. Firstly, 100g rubber powder was added to 500 g heated SK70 base asphalt (175 ± 5°C) and sheared for 40 min at 170°C in a high-shear mixer at 5000 rpm/min. Next 10g PPA was added to the blend and sheared for 20 min under the same shear conditions. Finally, the blend was developed for 30 min in an oven at 160°C to ensure the performance of final product.

## 3 Test methods

To explore the relationship between the micro technical indexes and the macro road performance of high modulus asphalt, rheological property test and molecular dynamics simulation of high modulus asphalts were performed. Rheological property testing was performed using a dynamic shear rheometer (Physical MCR101, manufactured by Antonpaar, Austria). Molecular dynamics simulations of asphalt were conducted using Materials Studio (MS) on the high-performance computing platform of Chang’an University.

### 3.1 Rheological property test

According to the test results of rheological properties of high modulus asphalt at the early stage of the research group [10], the shear modulus G*, rutting factor G*/sinδ and unrecoverable creep compliance (*J*_*nr*_) with good regularity were selected as the rheological properties indexes of correlation analysis in this study. The performance indexes are shown in Table 6.

**Table 6:** Performance indexes of high modulus asphalt.

Index	Recommended value
G* (60°C, 10Hz, 0.1%, KPa)	≥66.43
G* (60°C, 10Hz, 12%, KPa)	≥12.8
G*/sinδ (76°C, 5Hz, 0.1%, KPa)	≥7.03
J_nr,3.2_ (76°C, KPa^-1^)	≤2.53

The complex shear modulus G* and phase angle *δ* are the indexes used to characterize the high temperature performance of SHRP system. According to AASHTO MP1a-04 specification, G*/sinδ was not thought a good parameter to reflect the rutting resistance in many researches because that it did not take into account of recovery capacity of the modified asphalt. As G*/sinδ cannot effectively evaluate the high temperature performance of modified asphalt [11, 12], the repeated creep recovery test (MSCR) was adopted in NCHRP 9–10 which was proposed by the National Highway Cooperation Research Program of American. The repeated creep recovery test (MSCR) can judge the resistance to permanent deformation of asphalt, which is usually used to determine the elastic response of asphalt binder under shear creep and recovery at two stress levers. The unrecoverable creep compliance (*J*_*nr*_) is used as the main performance indicators, and the creep behavior can be used to investigate the rutting susceptibility of asphalt concrete [13–15]. The high modulus modified asphalt was subjected to Dynamic Shear Rheometer (DSR) tests at 60°C and 76°C.

### 3.2 Establishment of asphalt molecular model

**Fig 1:**
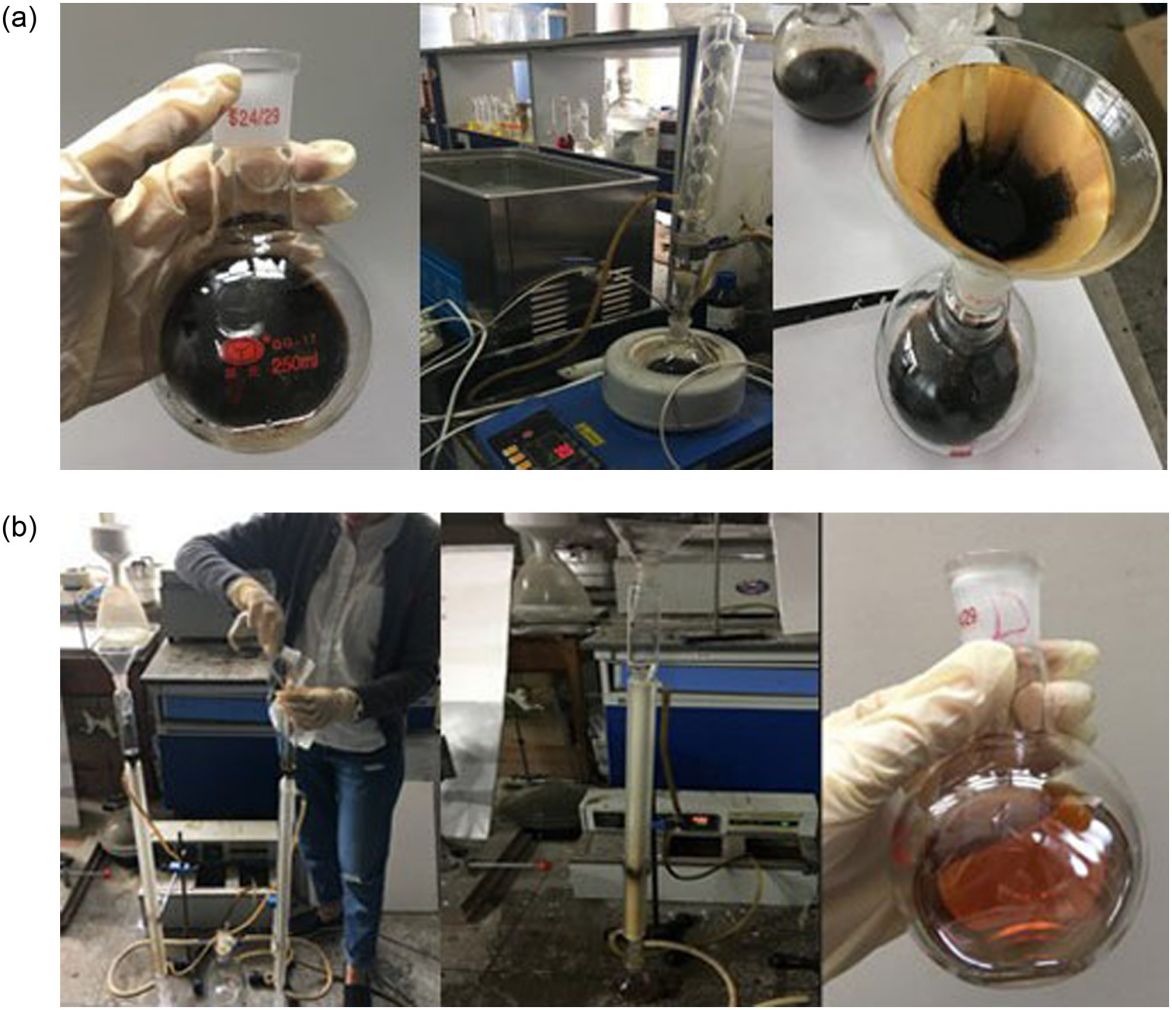
Asphalt four component separation test. (A) Solvent filtration method; (B) Chromatographic column separation method.

**Fig 2:**
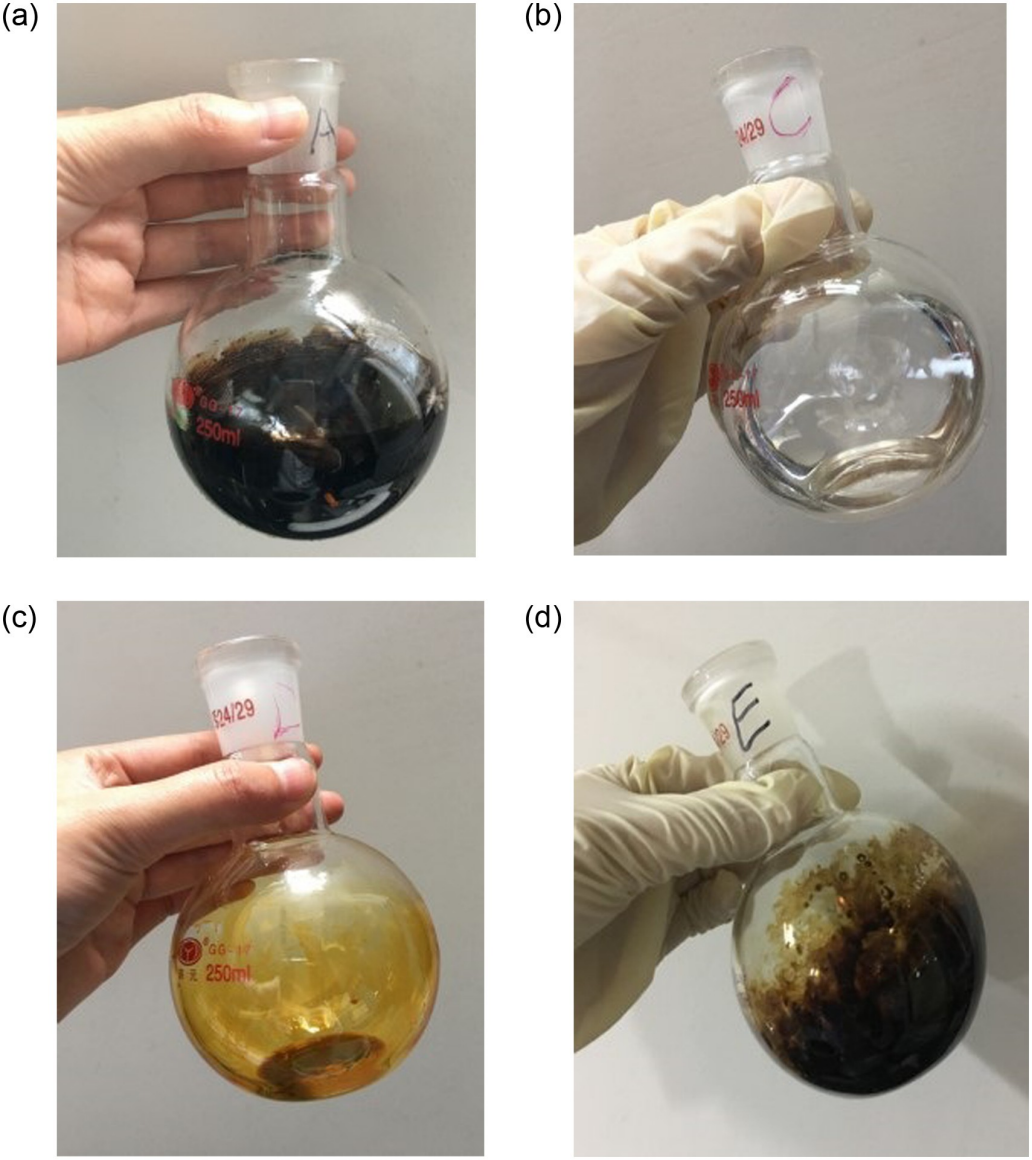
Separated four components of asphalt. (A) Asphaltene;(B) Saturates;(C) Aromatics;(D) Resin.

The component content and element composition of asphalt of asphalt was tested according to the specifications (JTG E20-2011) and specifications (GB T11132-2008), The separation of the four components was carried out using solvent filtration method and chromatographic column separation method according to the specifications, as shown in Fig 1. The separated four components are shown in Fig 2.

**Fig 3:**
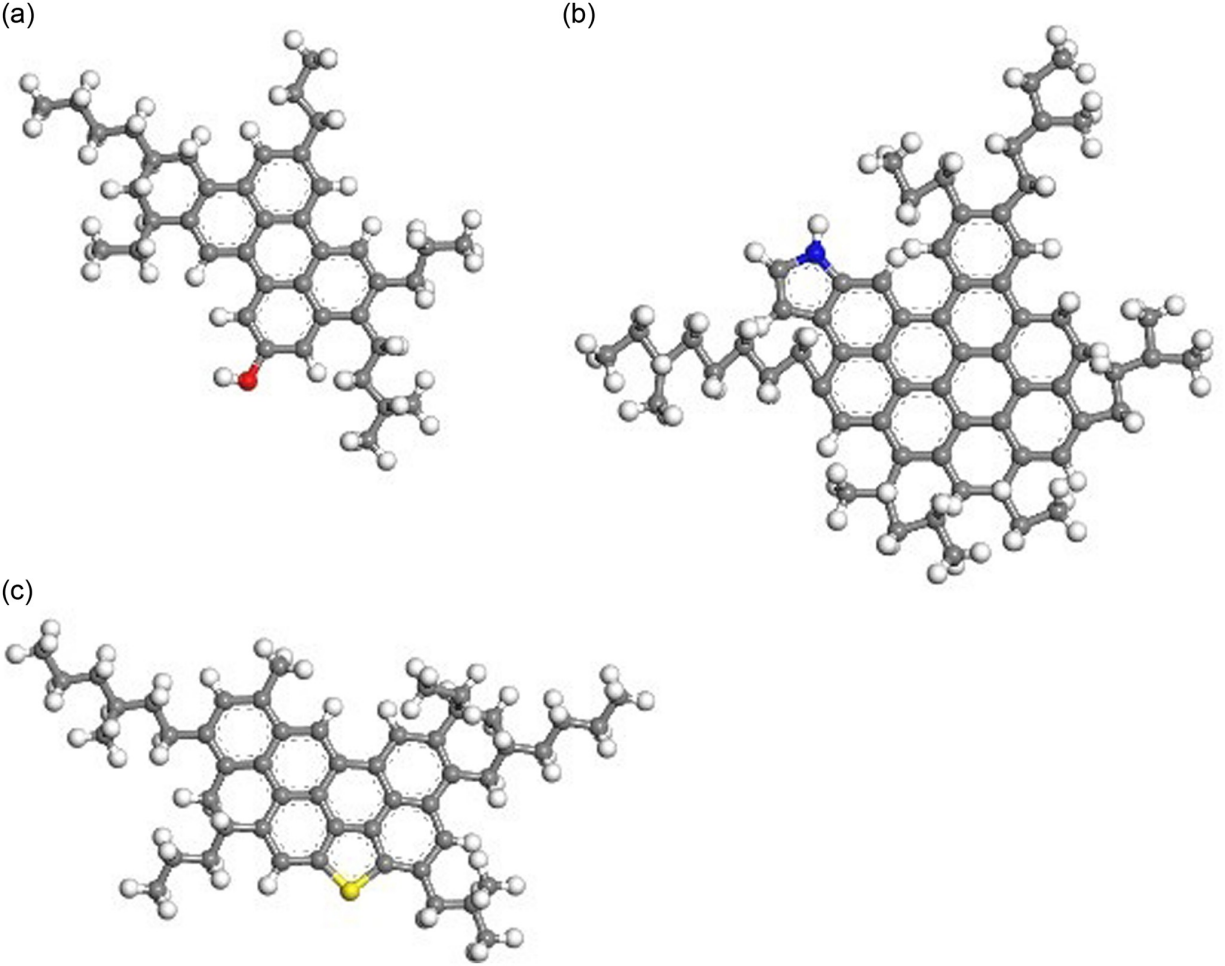
Representative molecular model of asphaltene. (A) Asphaltene 1;(B) Asphaltene 2; (C) Asphaltene 3.

**Fig 4:**
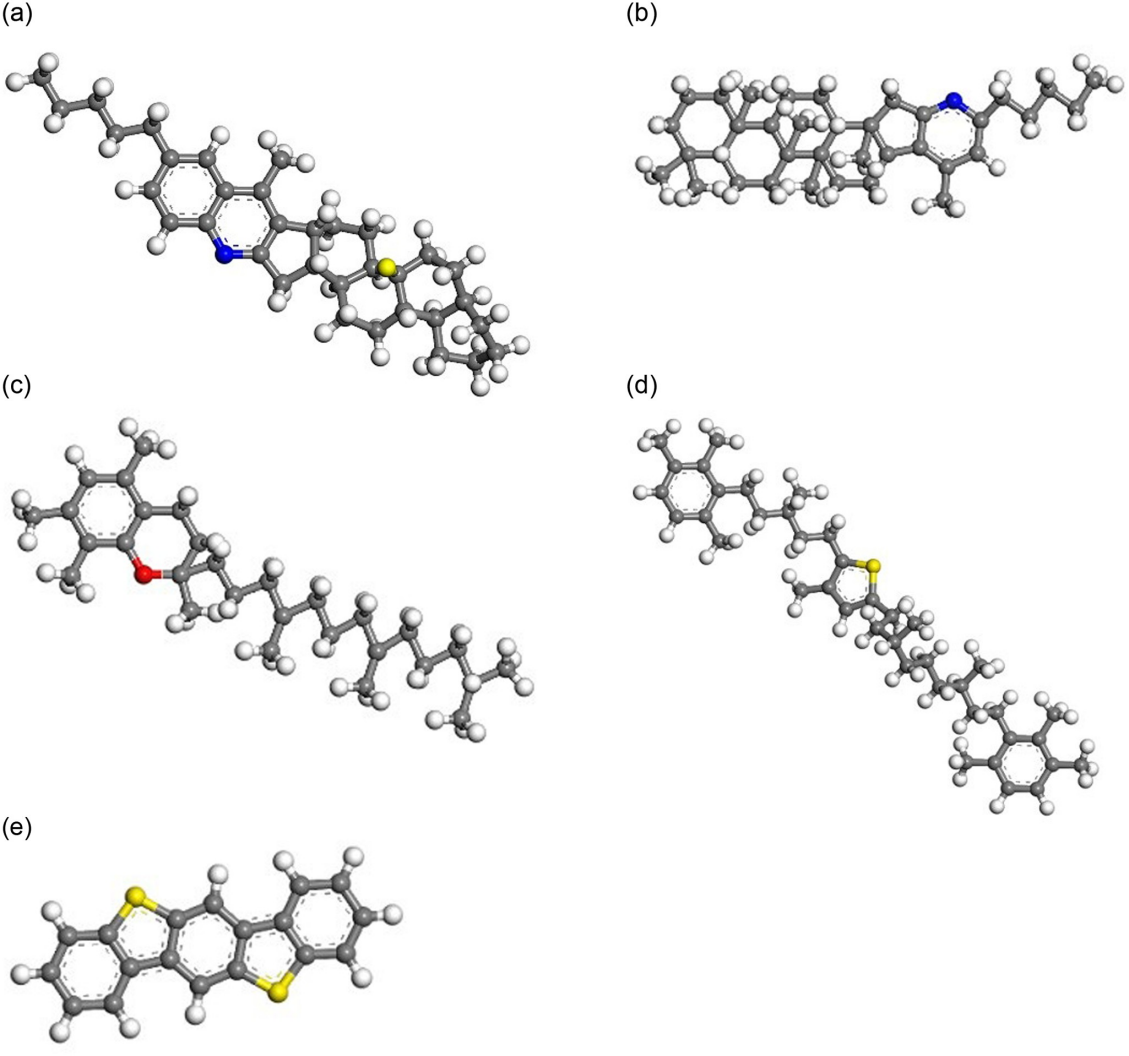
Representative molecular model of resin. (A) Resin 1;(B) Resin 2; (C) Resin 3; (D) Resin 4; (E) Resin 5.

**Fig 5:**
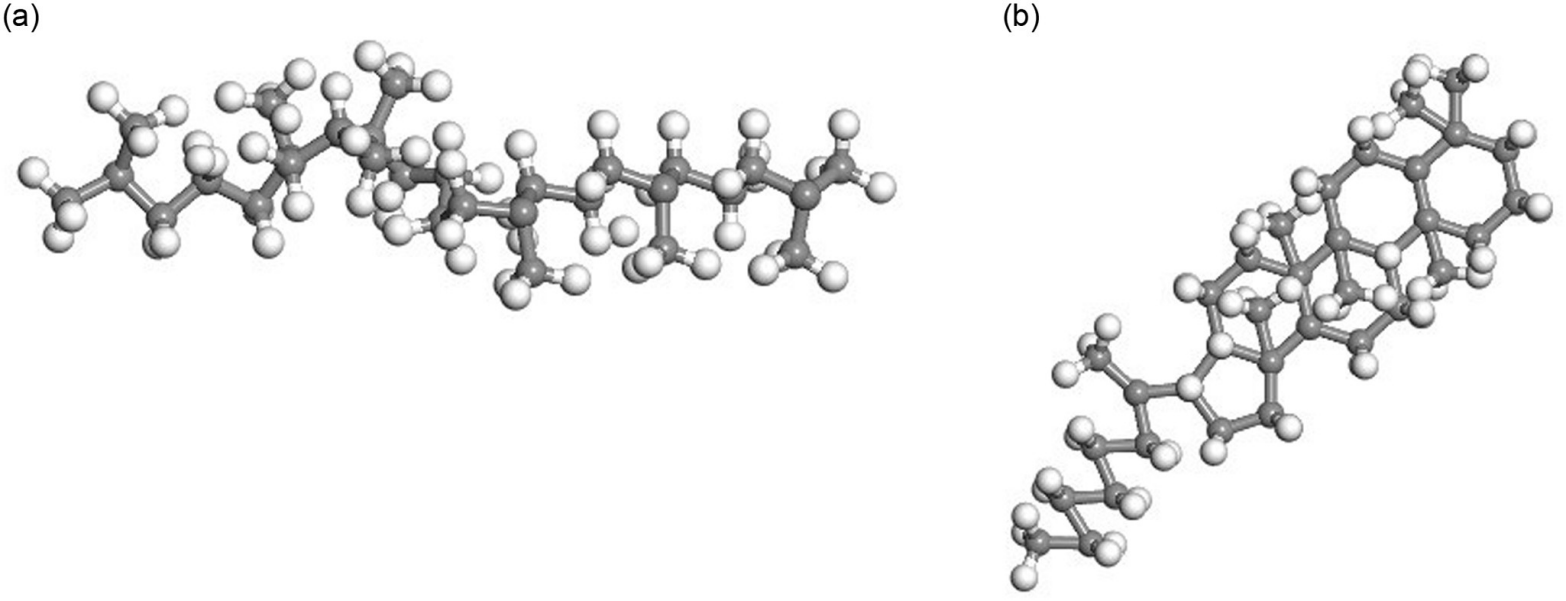
Representative molecular model of saturates. (A) Saturates 1; (B) Saturates 2.

**Fig 6:**
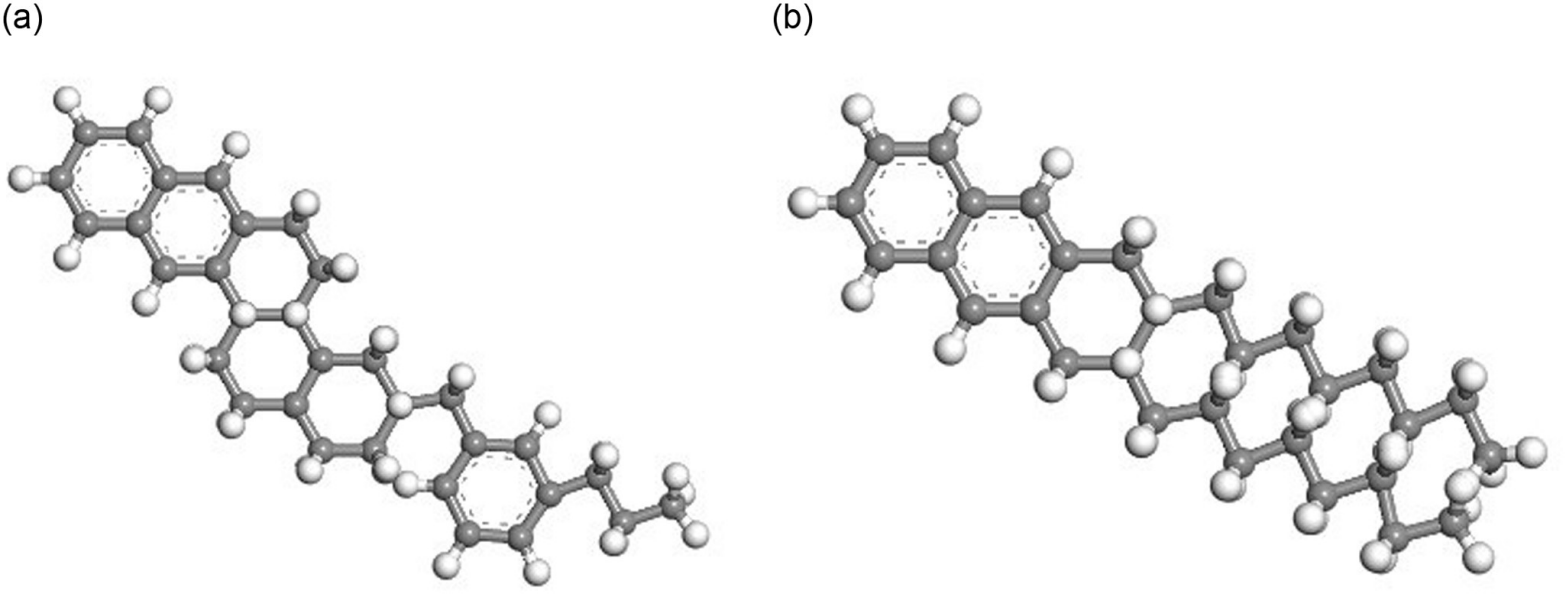
Representative molecular model of aromatics. (A)Aromatics 1; (B) Aromatics 2.

Then, the representative molecular of each component was selected to establish the asphalt molecular model based on existing research results [16–20], as shown in Figs 3–6.

Finally, the relative mass fractions of the main elements in the model were compared with the measured values in the elemental analysis test to verify the rationality of the four-component asphalt model, and based on the preliminary calculation results of the research group, the asphalt molecular model was assembled using the amorphous Amorphous Cell Calculate interface in MS. Firstly, the selected molecular structures of the four components of asphalt were randomly placed into a unit cell with a size of 39.7Å×39.7Å×39.7Å. Then, asphalt molecules were assembled at the interface of the amorphous “Amorphous Cell Calculate”, the task option was set to “Construction” in the interface parameter setting. Considering the computing power and accuracy of the server, “Quality” was set to “Fine”. In order to avoid large deviations in simulation results caused by the entanglement of molecular chains in the unit cell during the simulation process, the initial density of the simulation system was generally set to a smaller value. In this paper, the density was set to 0.8g/cm^3^. The electrostatic force and van der Waals interaction force were set to Ewald and Atom Baselaw, respectively. D. Calculate the force field using COMPASS force field, and establish a model after setting the parameters.

### 3.3 Molecular dynamics simulation

Firstly, combined with the results of asphalt molecular model, the molecular models of high modulus asphalt with different amounts of modifiers were assembled by Amorphous Cell Calculate interface in MS. Considering the computing power and precision of the server, Simulation quality is set to fine. In order to simulate the real state of material molecular movement as possible, Andersen and Berendsen methods [21, 22] were adopted for temperature and pressure control under periodic boundary conditions and Compass force fields, and The electrostatic and van der Waals forces were set to Ewald and Atom Based, respectively. After the parameters were set, the molecular model of the composite modified asphalt can be established. Then, the simulation system was subjected to 2000 steps of Geometry optimization by using the comprehensive method, and 100ps molecular dynamics simulation of the molecular system after the Geometry optimization was carried out under the NVT ensemble. That is, the model system in the state of energy stability was obtained. Finally, the physical modulus of high modulus asphalt molecular model in stable configuration was simulated at 333.15K (60°C) and 349.15K (76°C), respectively.

The Constant Strain method in the Forcite module was used to solve the physical modulus of the modified asphalt blend. After the dynamic superposition operation, the stiffness matrix and the flexibility matrix of the modified asphalt at 60°C and 76°C were obtained, as shown in Eq 1.

$$[c]=\left[\begin{array}{cccccc} \lambda +2u & \lambda & \lambda & 0 & 0 & 0\\ \lambda & \lambda +2u & \lambda & 0 & 0 & 0\\ \lambda & \lambda & \lambda +2u & 0 & 0 & 0\\ 0 & 0 & 0 & u & 0 & 0\\ 0 & 0 & 0 & 0 & u & 0\\ 0 & 0 & 0 & 0 & 0 & u \end{array}\right]$$ (1)

Where *λ* and *u* are lame constants; *c*_*ij*_ is stiffness constant.

Then the Young’s modulus (*E*), bulk modulus (*K*) and shear modulus (*G*) of high modulus asphalt were calculated by Formula (2)–(4) according to the stiffness matrix and flexibility matrix. The calculation formulas are as follows.

$$E=u\;\left(\frac{3\lambda +2u}{\lambda +u}\right)$$ (2)

$$K=\frac{f_{1}\left(c_{ij}\right)+f_{2}\left(s_{ij}\right)}{2}$$ (3)

$$G=\frac{f_{3}\left(c_{ij}\right)+f_{4}\left(s_{ij}\right)}{2}$$ (4)

Where *s*_*ij*_ is flexibility constant.

### 3.4 Correlation analysis and verification of micro index and macro index

According to the test results of rheological properties and the results of molecular dynamics simulation of LLDPE/SBS composite modified asphalt, the correlation between macro performance indexes and micro indexes was analyzed. Then through correlation analysis and regression calculation, the appropriate parameters of molecular dynamics simulation were obtained, and the estimation formula between the results of molecular dynamics simulation and high temperature rheological properties was established. Finally, the estimation formula was verified by combining the high temperature rheological test results and molecular dynamics simulation results of rubber/PPA composite modified asphalt.

## 4 Results and discussion

### 4.1 Test results of rheological properties

Six kinds of LLDPE/SBS composite modified asphalts with a total content (LLDPE: SBS in LLDPE/SBS composite modified asphalt is 1:1) of modifiers of 6%, 7%, 8%, 9%, 10%, and 11% were selected for rheological performance testing. The test results are shown in Table 7.

**Table 7:** Rheological properties test results of LLDPE/SBS composite modified asphalt.

Total content (%)	60°C, 10Hz (Pa)		76°C, 5Hz	MSCR (76°C)
	G* (0.1%)	G* (12%)	G*/sinδ (Pa)	J_nr,3.2_ (KPa^-1^)
6	50260	47674	5877	4.8241
7	53100	51306	6978	3.6729
8	65365	62528	8608	2.5756
9	94975	88089	13592	1.6212
10	102864	92056	14463	1.5260
11	103490	97120	14595	1.4522
Requirement	≧60000	≧10000	≧5000	≦2

It can be seen from Table 7 that the rutting factor and shear modulus of the composite modified asphalt increase with the increase of the total content of LLDPE and star SBS, and the unrecoverable creep compliance decreases, which indicates that the high temperature performance of the composite modified asphalt increases with the increase of the modifier. When the total content of the modifier reaches 9%, the high temperature performance of LLDPE/SBS composite modified asphalt meets the requirements of high modulus asphalt.

### 4.2 Assembly of asphalt molecular model

The results of the four-component separation test of SK70 asphalt are shown in Table 8.

**Table 8:** Results of the four-component separation test of SK70 asphalt.

Total mass	Asphalt	Asphaltene	Saturates	Aromatics	Resin
Flask (g)	143.005	143.016	149.401	148.090	149.947
Components (g)	1.01	0.142	0.182	0.385	0.300
Content (%)	-	14.059	18.020	38.119	29.703

According to the test results of asphalt components in Table 8 and the test results of asphalt chemical components in earlier stage of this paper [23], the molecular structure of each component of asphalt were selected combined with the existing research results and the trial results of the atoms number allowed in the simulation system, as shown in Table 9.

**Table 9:** Selection results of representative molecular structure of asphalt.

Molecule	Chemical formula	Atomic number	Molecular weight
Asphaltene 1	C_42_H_54_O	97	574
Asphaltene 2	C_66_H_81_N	148	887
Asphaltene 3	C_51_H_62_S	114	706
Resin 1	C_40_H_59_N	100	553
Resin 2	C_36_H_57_N	94	503
Resin 3	C_29_H_50_O	80	414
Resin 4	C_40_H_60_S	101	572
Resin 5	C_18_H_10_S_2_	30	290
Saturates 1	C_30_H_62_	92	422
Saturates 2	C_35_H_62_	97	482
Aromatics 1	C_35_H_44_	79	464
Aromatics 2	C_30_H_46_	76	406

The content of each component of the asphalt was calculated according to the molecular composition of the asphalt molecular model in Table 9, and compared with the measured values, the comparison results are shown in Table 10.

**Table 10:** Comparison of simulated and measured values of the component content of SK70 asphalt.

Component	Simulated value (%)	Measured value (%)
Asphaltene	13.436	14.059
Resin	29.601	29.703
Saturates	18.124	18.020
Aromatics	38.839	38.119

Table 10 shows that compared with the measured value, the four-component content of the asphalt model system obtained through trial calculation is slightly higher, but the proportion of wax content in the asphalt meets the specification requirements, so the impact of error on the asphalt performance can also be ignored.

In order to verify the rationality of the asphalt model structure, the relative mass fraction of each element in the asphalt model system is calculated and compared with the measured value of the relative mass fraction of elements in the element analysis test. The comparison results are shown in Table 11.

**Table 11:** Comparison of calculated and measured values of relative mass fraction of elements.

Value	C (%)	H (%)	O (%)	N (%)	S (%)
Simulated value	86.862	10.709	0.397	0.347	1.686
Measured value	85.930	11.526	0.382	0.356	1.780
Relative error	1.085	7.088	3.927	2.528	5.281

It can be seen from Table 11 that the relative error between the relative mass fraction of each element in the four-component model established by the assembly method and the measured results in the element analysis test is less than 10%, and the effect of this error range on the molecular dynamics simulation of asphalt can be ignored.

**Fig 7:**
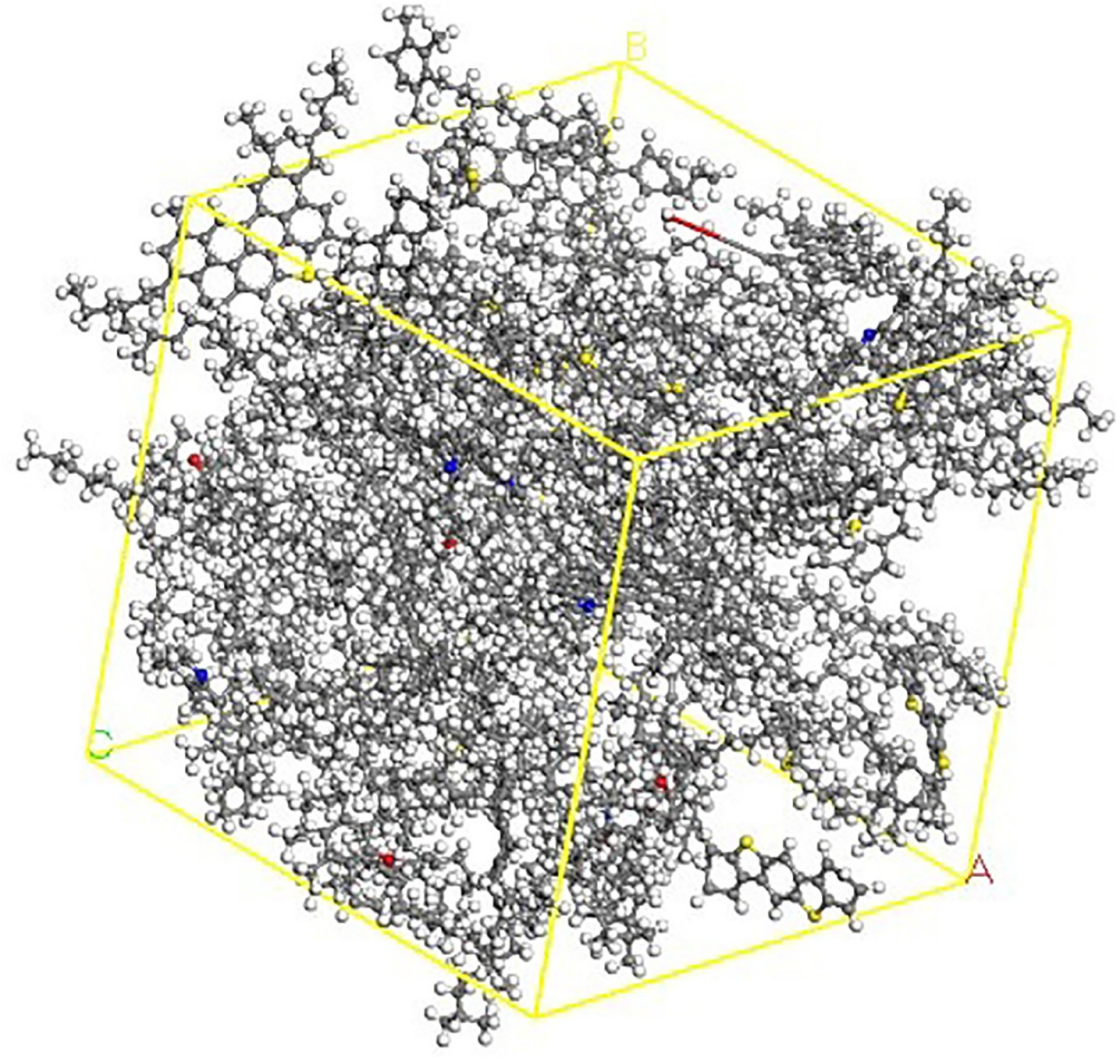
Four-component molecular model of asphalt.

According to the composition of asphalt in Table 9, the asphalt molecular model was assembled through the Amorphous Cell Calculate interface in MS, as shown in Fig 7.

### 4.3 Simulation results of physical modulus

**Fig 8:**
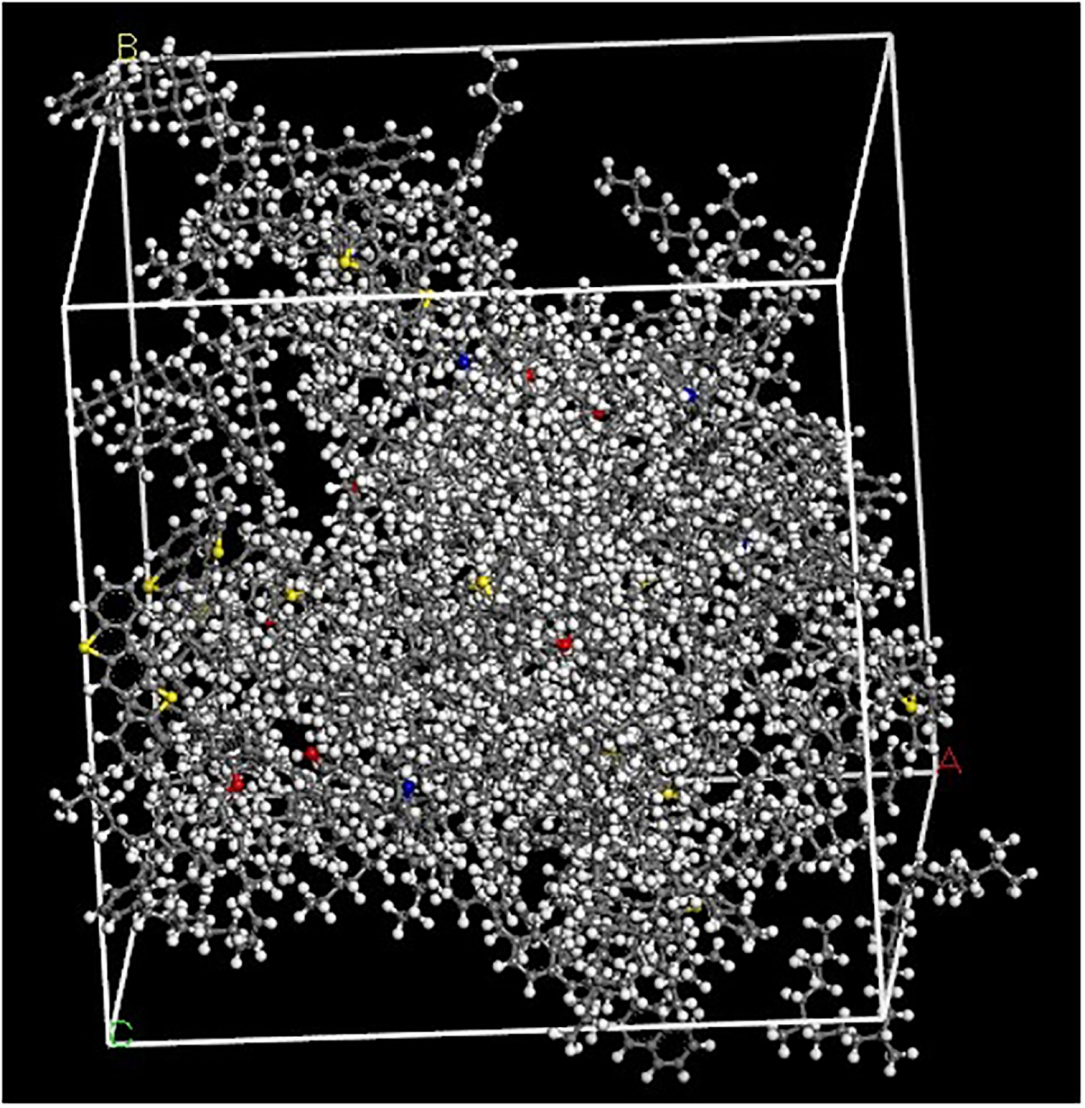
Blending system model of LLDPE / SBS composite modified asphalt.

Combined with the four component asphalt molecular model, taking the modified asphalt with the minimum content (the total content is 6%) in Table 7 as an example, the blending system model of LLDPE/SBS composite modified asphalt is as shown in Fig 8.

**Fig 9:**
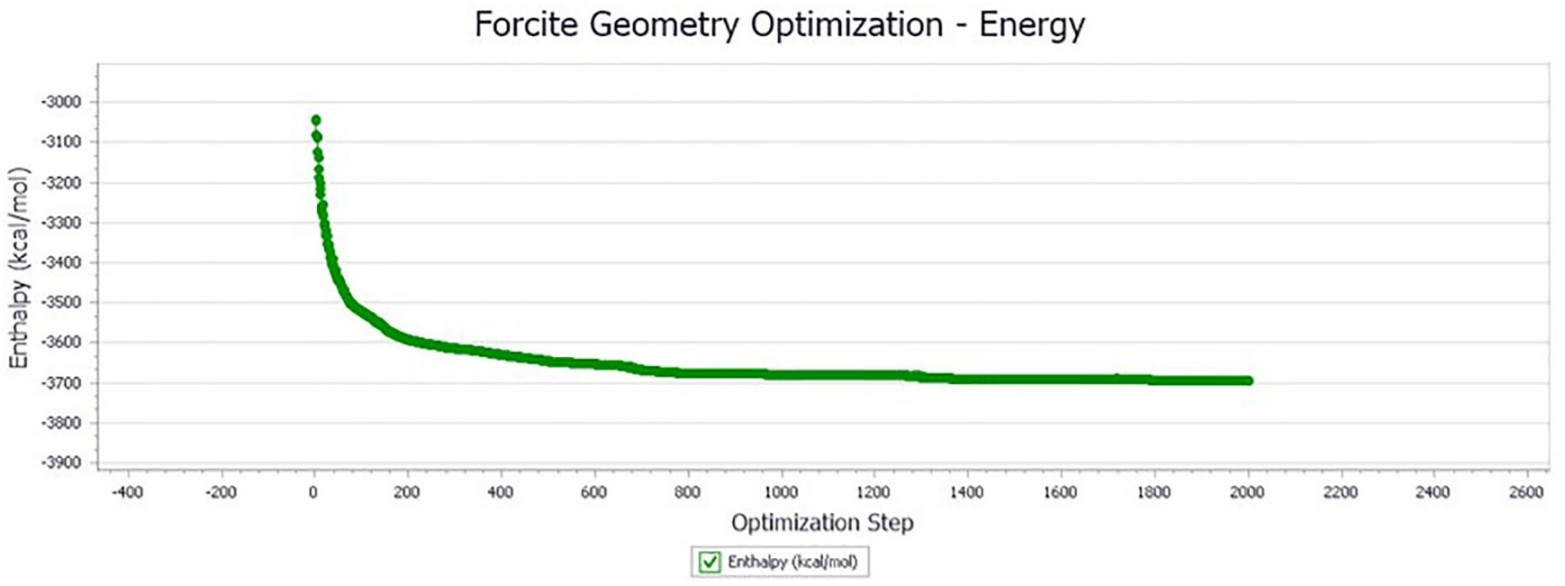
Geometry optimization result of LLDPE/SBS composite modified asphalt blend system.

The initially constructed blend system model is in a high-energy state, which is easy to cause the discreteness and distortion of the molecular dynamics simulation results. The Geometry optimization of the high-energy unsteady simulation system was carried out by the comprehensive method, and the result is shown in Fig 9.

**Fig 10:**
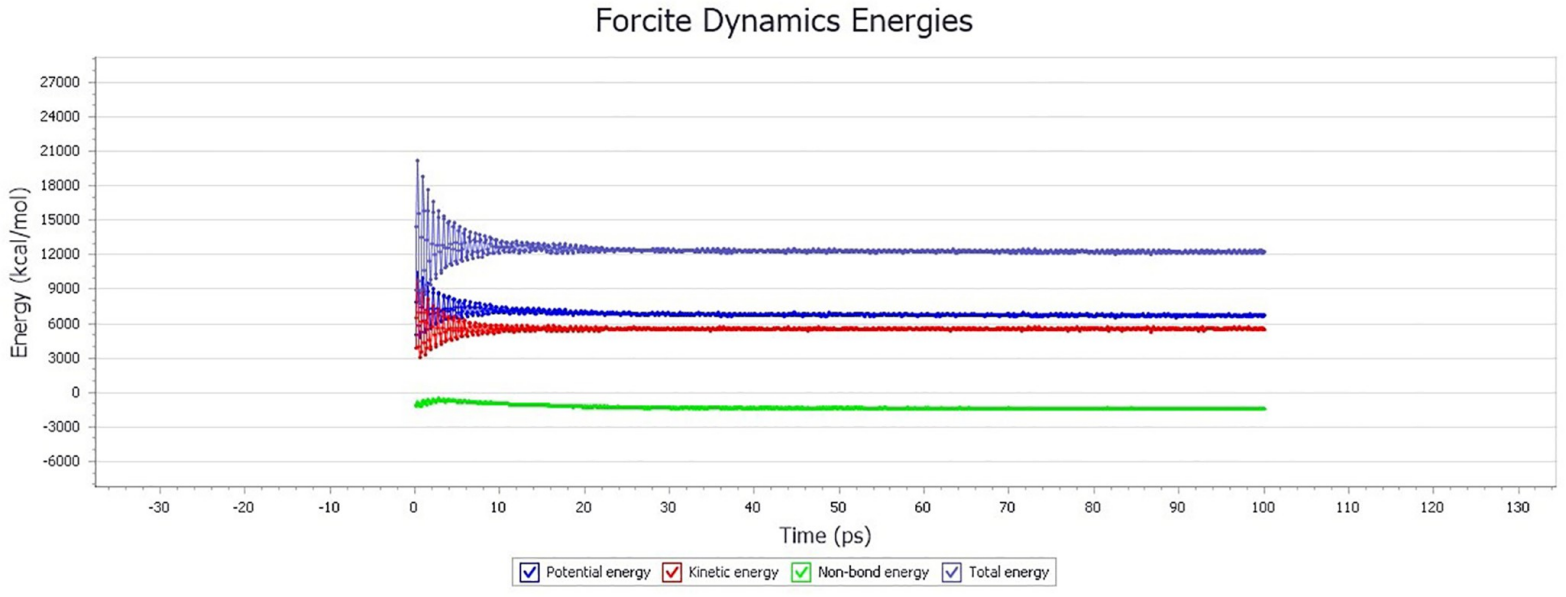
Molecular dynamics simulation result of LLDPE/SBS composite modified asphalt.

Fig 9 shows that with the increase of the optimization step, the total potential energy of the simulation system gradually decreases and tends to be gentle after the Geometry optimization process. In order to further improve the simulation accuracy and make the simulation system approach the actual structure of the material to the largest extent, the molecular dynamics simulation of the molecular model after the Geometry optimization was carried out. The molecular dynamics simulation result is shown in Fig 10.

According to the judgment standard for stable state of molecular dynamics simulation system [23], when the energy-time curve tends to be stable and the fluctuation presents regular changes, it can be determined that the system reaches the energy stable state, that is it reaches the termination condition of dynamics calculation before the simulation analysis of molecular dynamics performance. The total energy of the system is the sum of the total potential energy and the total kinetic energy of the system. From Fig 10, it can be seen that the change rule of the total potential energy and the total kinetic energy of the system is basically the same as the change rule of the total energy through molecular dynamics simulation. After 10ps molecular dynamics calculation, the total kinetic energy, the total potential energy, the total energy and the non-bonding energy of the system tend to be stable. That is to say, the simulation system has reached an energy stable state, and the structure system is in a stable state after 10ps molecular dynamics simulation, which can be further used in molecular dynamics simulation calculation and analysis.

The physical modulus of LLDPE/SBS composite modified asphalt stabilized system after molecular dynamics simulation was simulated by the mechanical properties command of the Force module in MS, and the simulation was carried out at 60°C and 76°C respectively. The results of simulation are shown in Tables 12–15.

**Table 12:** Stiffness matrix of LLDPE/SBS composite modified asphalt (60°C).

*c*_*ij*_	C_1_	C_2_	C_3_	C_4_	C_5_	C_6_
R_1_	2.5485	4.0568	7.2669	2.1302	3.0070	6.4408
R_2_	4.0568	1.5085	8.5390	5.3918	3.0266	3.2686
R_3_	7.2669	8.5390	3.4100	7.4703	4.6233	3.9451
R_4_	2.1302	5.3918	7.4703	10.6583	4.6769	5.1844
R_5_	3.0070	3.0266	4.6233	4.6769	0.4086	2.3316
R_6_	6.4408	3.2686	3.9451	5.1844	2.3316	5.2817

**Table 13:** Flexibility matrix of LLDPE/SBS composite modified asphalt (60°C).

*s*_*ij*_	C_1_	C_2_	C_3_	C_4_	C_5_	C_6_
R_1_	355.3632	808.8667	445.2391	227.1769	918.1805	81.8309
R_2_	808.8667	619.0037	208.0147	660.9763	1343.8582	921.4399
R_3_	445.2391	208.0147	1112.0090	551.2752	1498.5017	834.3866
R_4_	227.1769	660.9763	551.2752	436.2006	2118.0268	759.5906
R_5_	918.1805	1343.8582	1498.5017	2118.0268	2637.5765	432.3951
R_6_	81.8309	921.4399	834.3866	759.5906	432.3951	1144.2481

**Table 14:** Stiffness matrix of LLDPE/SBS composite modified asphalt (76°C).

*c*_*ij*_	C_1_	C_2_	C_3_	C_4_	C_5_	C_6_
R_1_	1.4388	4.1138	2.9649	0.6679	1.6452	5.6759
R_2_	4.1138	1.6407	4.0831	1.2032	0.5482	2.8193
R_3_	2.9649	4.0831	6.3946	2.0042	0.9757	0.7949
R_4_	0.6679	1.2032	2.0042	2.8293	4.5666	1.0458
R_5_	1.6452	0.5482	0.9757	4.5666	3.1242	0.5391
R_6_	5.6759	2.8193	0.7949	1.0458	0.5391	1.1736

**Table 15:** Flexibility matrix of LLDPE/SBS composite modified asphalt (76°C).

*s*_*ij*_	C_1_	C_2_	C_3_	C_4_	C_5_	C_6_
R_1_	264.3634	573.9443	361.6661	295.3833	631.7892	118.8474
R_2_	573.9443	160.8586	471.4138	1028.6421	255.6434	999.4657
R_3_	361.6661	471.4138	707.1716	610.8728	643.0011	382.3943
R_4_	295.3833	1028.6421	610.8728	1384.6174	828.2771	304.7352
R_5_	631.7892	255.6434	643.0011	828.2771	142.1160	360.0298
R_6_	118.8474	999.4657	382.3943	304.7352	360.0298	722.7952

By the same way, the model establishment and molecular dynamics calculation of LLDPE/SBS composite modified asphalt with different content of modifier were carried out. The stiffness matrix and flexibility matrix of high modulus composite modified asphalt with different content of modifier at 60°C and 76°C were obtained. Then, the physical modulus molecular dynamics simulation results of LLDPE/SBS composite modified asphalt at two temperatures were calculated by Formula (2)–(4), as shown in Table 16.

**Table 16:** Simulation results of physical modulus of LLDPE/SBS.

Total content(%)	60°C (MPa)			76°C (MPa)		
	E	K	G	E	K	G
6	14375.9	16237.2	722.9	12128.5	14401.8	631.4
7	14580.6	17043.3	734.5	12908.6	14887.9	655.3
8	15828.7	17502.5	761.8	13938.7	15693.2	673.1
9	15894.1	17886.9	777.2	14039.2	16267.7	713.8
10	16474.7	17949.7	782.1	14275.4	16711.4	725.3
11	16531.4	18239.2	786.0	14356.5	16842.0	727.9

From the molecular dynamics simulation results in Table 16, it can be seen that the simulation results of modulus are quite different from the data measured in the laboratory, but with the increase of the total content of modifier, the molecular dynamics simulation results of shear modulus, bulk modulus and Young’s modulus of LLDPE/SBS composite modified asphalt increase, which is consistent with the change trend of the test results measured in Table 7. This shows that for the physical modulus, there is a certain correlation between the simulation results and the measured data in laboratory of LLDPE/SBS composite modified asphalt.

### 4.4 Correlation analysis and verification results of micro and macro indexes

**Fig 11:**
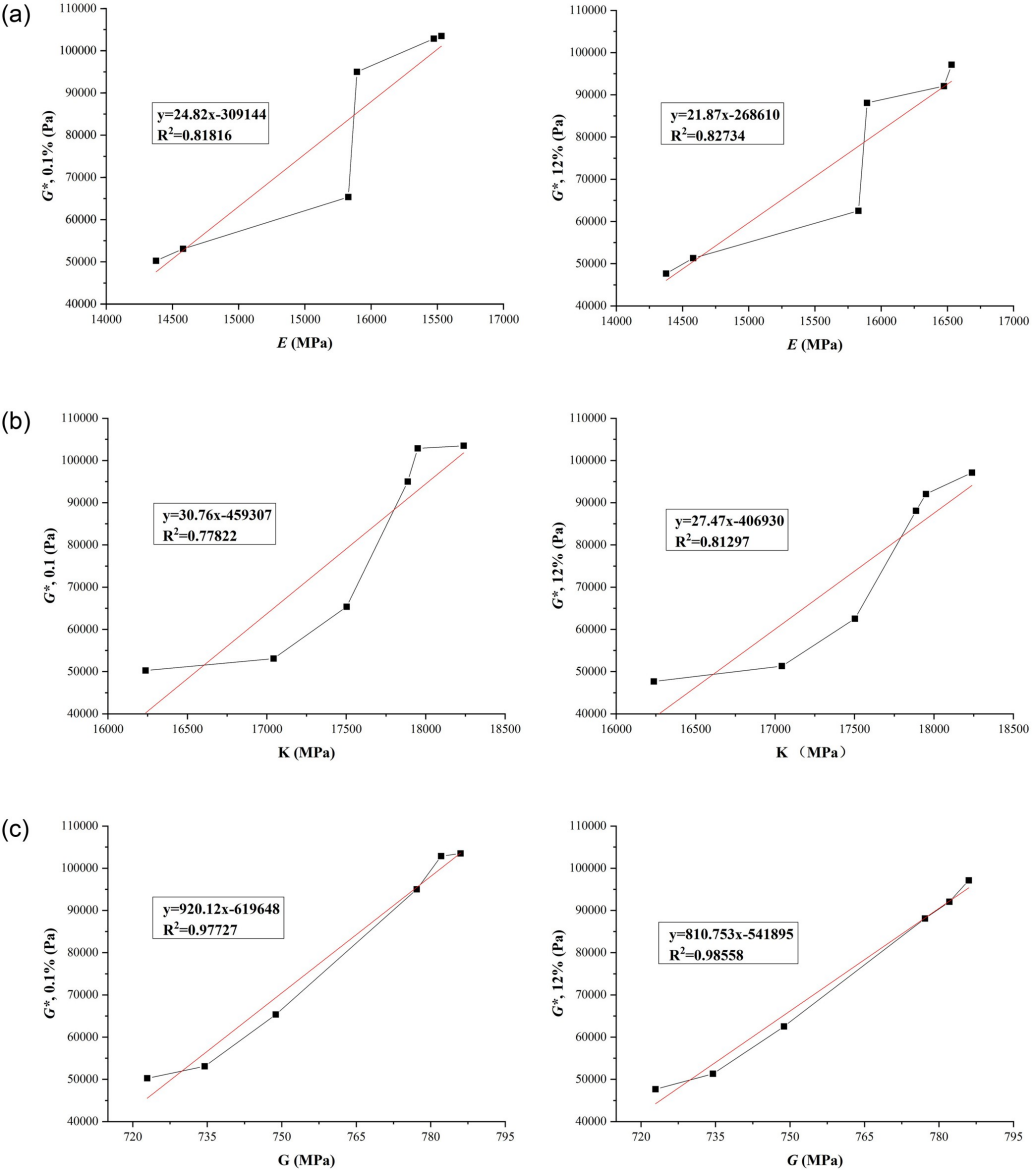
Linear fitting results of micro and macro indexes of LLDPE/SBS composite modified asphalt (60°C). (A) Young’s modulus-rheological property index; (B) Bulk modulus-rheological property index; (C) Shear modulus-rheological property index.

**Fig 12:**
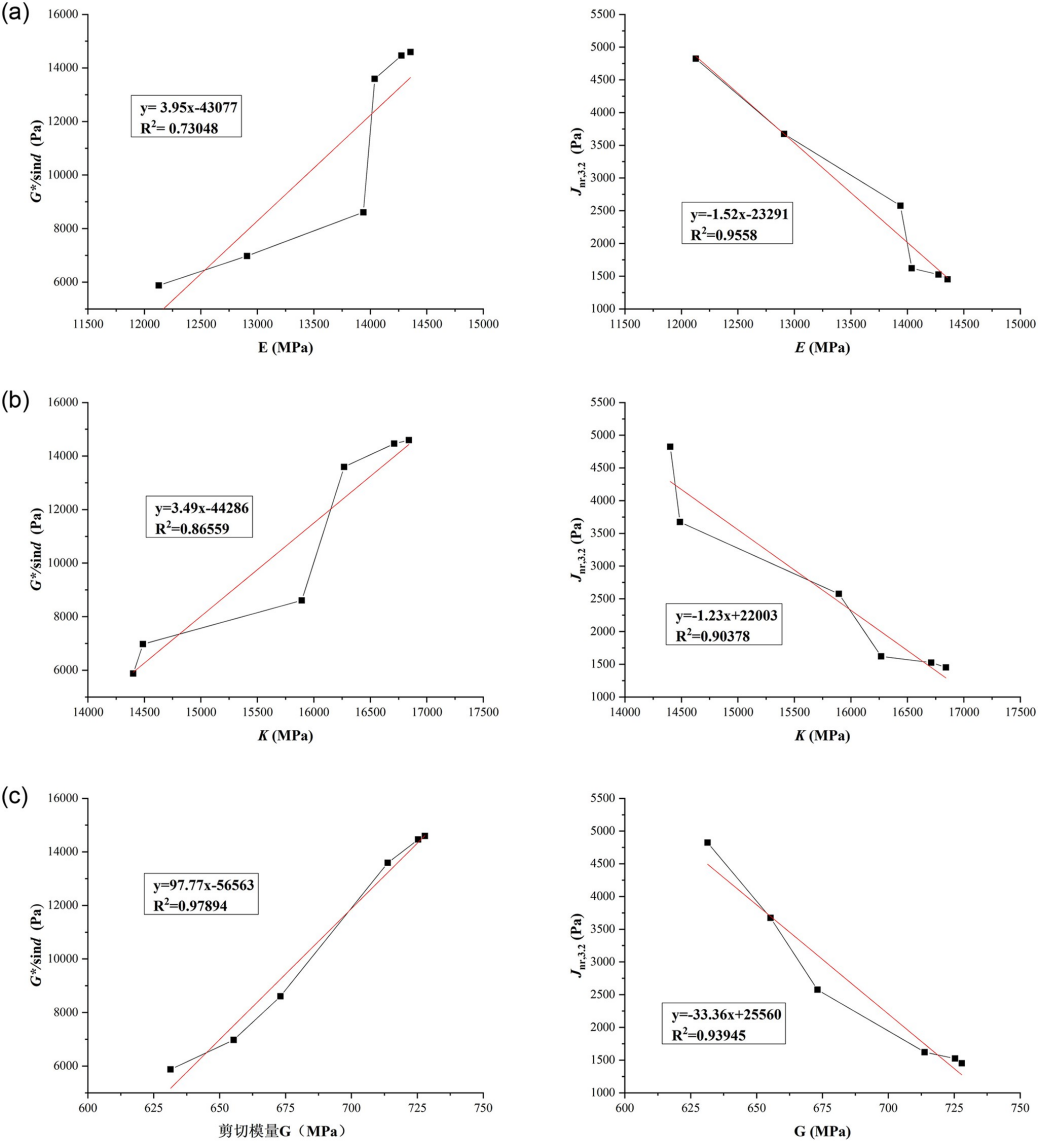
Linear fitting results of micro and macro indexes of LLDPE/SBS composite modified asphalt (76°C). (A) Young’s modulus-rheological property index; (B) Bulk modulus-rheological property index; (C) Shear modulus-rheological property index.

According to the rheological performance test results and physical modulus molecular dynamics simulation results of the LLDPE/SBS composite modified asphalt in Tables 7 and 16, the correlation analysis between the macro and micro indexes was obtained. The linear fitting results of the modulus simulation results and the rheological performance test results of the LLDPE/SBS composite modified asphalt at different modifier content and temperature are shown in Figs 11 and 12.

Figs 11 and 12 show that the correlation between micro and macro indexes of LLDPE/SBS composite modified asphalt is relatively high. And the correlation coefficient statistical results of micro and macro indexes are shown in Table 17.

**Table 17:** Correlation coefficient (R^2^) between micro and macro indexes of LLDPE/SBS composite modified asphalt.

Physical modulus	R^2^, 60°C		R^2^, 76°C	
	G* (0.1%)	G* (12%)	G*/sinδ	J_nr,3.2_
E	0.81816	0.82734	0.73048	0.9558
K	0.77822	0.81297	0.86559	0.90378
G	0.97727	0.98558	0.97894	0.93945

The correlation coefficient of linear fitting is above 0.9, which indicates that the correlation between the two indexes is good. Table 17 shows among the three modulus, only the correlation coefficient of shear modulus between the molecular dynamics simulation results and the rheological property test results is above 0.9, which not only shows that the modified asphalt can be effectively simulated and evaluated by molecular dynamics simulation, but also shows that the shear modulus is a high temperature index with a good correlation between molecular dynamics simulation results and rheological test results. Thus the estimation formulas between molecular dynamics simulation indexes and high temperature rheological performance indexes were established according to the linear fitting results of micro and macro indexes of High modulus asphalt, the estimation formula is shown in Formula (5)–(8).

$$y_{G*,0.1\%\;(60{}^{\circ}\text{C})}=920.12x_{G}-619648$$ (5)

$$y_{G*,12\%\;(60{}^{\circ}\text{C})}=810.75x_{G}-541895$$ (6)

$$y_{G*/\;\text{sin}\;\delta (76{}^{\circ}\text{C})}=97.77x_{G}-56563$$ (7)

$$y_{J_{\text{nr},3.2}(76{}^{\circ}\text{C})}=-33.36x_{G}+25560$$ (8)

Where *x*_*G*_ is the result of molecular dynamics simulation calculation of shear modulus, MPa; *y* is the result of rheological property test, Pa.

To further verify the accuracy of the estimation formulas, the other modified asphalt, the rubber/PPA high modulus asphalt was subjected to molecular dynamics simulation of shear modulus and rheological properties test. Herein the Rubber asphalt (20% rubber) was modified by PPA with a content of 1%, 1.5%, 2%, 2.5%, and 3% respectively. Molecular models of rubber/PPA high modulus asphalt with different modifier content were established, and the shear modulus of different molecular models was simulated by the Constant Strain method in the Forcite module. The simulation results are shown in Table 18.

**Table 18:** Molecular dynamics simulation results of shear modulus of rubber/PPA composite modified asphalt.

Shear modulus		PPA content (%)				
		1.00	1.50	2.00	2.50	3.00
G (MPa)	60°C	715.4	720.6	739.8	745.3	754.1
	76°C	677.2	690.2	711.5	721.8	733.7

It can be seen from Table 18, with the increase of PPA content, the results of molecular dynamics simulation results of shear modulus of rubber/PPA high modulus asphalt gradually increase, while with the increase of simulation temperature, the shear modulus shows the opposite trend, has the same trend as laboratory test results.

According to the estimation formula of Formula (5)–(8), combined with the molecular dynamics simulation results of the shear modulus in Table 18, the estimated value of rheological properties of rubber/PPA composite modified asphalt with different PPA content is shown in Table 19.

**Table 19:** Estimated value of rheological properties of rubber/PPA high modulus asphalt.

PPA content (%)	60°C, 10Hz (Pa)		76°C, 5Hz G*/sinδ (Pa)	MSCR (76°C)
	G* (0.1%)	G* (12%)		J_nr,3.2_ (KPa^-1^)
1.00	38606	38115	9647	2.9686
1.50	43390	42331	10918	2.5349
2.00	61057	57898	13000	1.8244
2.50	66117	62357	14007	1.4808
3.00	71214	69492	15170	1.0838

In order to verify the accuracy of the estimation formulas of micro and macro indexes, the rheological properties were tested, and the test results are shown in Table 20.

**Table 20:** Test results of rheological properties of rubber/PPA high modulus asphalt.

PPA content (%)	60°C, 10Hz (Pa)		76°C, 5Hz G*/sinδ (Pa)	MSCR (76°C)
	G* (0.1%)	G* (12%)		J_nr,3.2_ (KPa^-1^)
1.00	39221	38008	9493	2.9619
1.50	44437	41127	10625	2.5327
2.00	60475	54768	13105	1.8529
2.50	66503	59544	14532	1.5178
3.00	75868	68719	15314	1.1255

The estimation value of rheological property in Table 19 was compared with the measured value in Table 20, and the relative error between the estimation value and the measured value is shown in Table 21.

**Table 21:** Relative error between estimation value and the measured value (%).

PPA content (%)	60°C, 10Hz (Pa)		76°C, 5Hz G*/sinδ (Pa)	MSCR (76°C)
	G* (0.1%)	G* (12%)		J_nr,3.2_ (KPa^-1^)
1.00	1.57	0.28	1.62	0.23
1.50	2.36	2.93	2.76	0.09
2.00	0.96	5.72	0.80	1.54
2.50	0.58	4.72	3.61	2.44
3.00	6.13	1.12	0.94	3.71

Table 21 shows that the relative error between the measured value and the estimation value of high temperature parameters were obtained from the estimation formulas of micro and macro indexes of the high modulus asphalt is less than 7%, and the relative error of most of the indexes is less than 3%. It can be seen that the high temperature parameters obtained by molecular dynamics simulation can predict the high temperature performance of high modulus modified asphalt to a certain extent.

## 5 Conclusions

The molecular dynamics simulation results of modulus are different from the data measured in the laboratory, but the molecular dynamics simulation results of shear modulus, bulk modulus and Young’s modulus of high modulus asphalt are consistent with the change trend of the test results. This shows that, for LLDPE/SBS composite modified asphalt, there is a certain correlation between the simulation results of the physical modulus and the measured data in laboratory.

The estimation formulas of macro and micro indexes of the high modulus modified asphalt were established according to the correlation analysis between the rheological performance test results and physical modulus molecular dynamics simulation result. And among the three modulus, only the correlation coefficient of shear modulus is above 0.9, which shows that the shear modulus is a high temperature index, showing a good correlation between molecular dynamics simulation results and rheological test results.

For the high modulus asphalt, the relative error between the measured value and the estimation value of high temperature parameters were obtained from the estimation formulas of micro and macro indexes is less than 7%, and the relative error of most of the indexes is less than 3%, which show that the high temperature parameters obtained by molecular dynamics simulation can predict the high temperature performance of high modulus modified asphalt under the limited experimental conditions.

Molecular dynamics is an important simulation characterization method and is very helpful for simulating and predicting various properties of asphalt. The research results of this paper can effectively predict the high-temperature performance of high modulus asphalt, which has important reference value for improving the design level of high modulus modified asphalt and its mixtures, and have important theoretical guidance significance for the promotion and application of polymer modified asphalt. However, there are still some differences between the model established in the paper and the model of high modulus asphalt. In the future, the model will be further optimized to study the modification mechanism and mechanical properties of high modulus asphalt.
